# Molecular basis of the phenotypic variants arising from a *Pseudoalteromonas lipolytica* mutator

**DOI:** 10.1099/mgen.0.001118

**Published:** 2023-10-18

**Authors:** Zhenshun Zeng, Jiayu Gu, Shituan Lin, Qian Li, Weiquan Wang, Yuexue Guo

**Affiliations:** ^1^​ Key Laboratory for Water Quality and Conservation of the Pearl River Delta, Ministry of Education, School of Environmental Science and Engineering, Guangzhou University, Guangzhou, PR China; ^2^​ Key Laboratory of Tropical Marine Bio-Resources and Ecology, Guangdong Key Laboratory of Marine Materia Medica, RNAM Center for Marine Microbiology, South China Sea Institute of Oceanology, Chinese Academy of Sciences, Guangzhou, PR China; ^3^​ University of Chinese Academy of Sciences, Beijing, PR China

**Keywords:** capsular polysaccharide, genome resequencing, mismatch repair system, mutator, *Pseudoalteromonas*

## Abstract

Bacterial deficiencies in the DNA repair system can produce mutator strains that promote adaptive microevolution. However, the role of mutator strains in marine *Pseudoalteromonas *, capable of generating various gain-of-function genetic variants within biofilms, remains largely unknown. In this study, inactivation of *mutS* in *Pseudoalteromonas lipolytica* conferred an approximately 100-fold increased resistance to various antibiotics, including ciprofloxacin, rifampicin and aminoglycoside. Furthermore, the mutator of *P. lipolytica* generated variants that displayed enhanced biofilm formation but reduced swimming motility, indicating a high phenotypic diversity within the Δ*mutS* population. Additionally, we observed a significant production rate of approximately 50 % for the translucent variants, which play important roles in biofilm formation, when the Δ*mutS* strain was cultured on agar plates or under shaking conditions. Using whole-genome deep-sequencing combined with genetic manipulation, we demonstrated that point mutations in *AT00_17115* within the capsular biosynthesis cluster were responsible for the generation of translucent variants in the Δ*mutS* subpopulation, while mutations in flagellar genes *fliI* and *flgP* led to a decrease in swimming motility. Collectively, this study reveals a specific mutator-driven evolution in *P. lipolytica *, characterized by substantial genetic and phenotypic diversification, thereby offering a reservoir of genetic attributes associated with microbial fitness.

## Data Summary

The whole-genome resequencing data have been deposited in the Sequence Read Archive at NCBI under Bioproject accessions PRJNA739013 and PRJNA805367 for the wild-type and Δ*mutS* samples, respectively.

**Impact Statement**

Mutator strains are commonly found in clinical and experimental populations of model pathogens, and facilitate rapid diversification through increased genomic mutations. However, our understanding of mutator-driven diversification in marine bacteria remains limited. *Pseudoalteromonas *, an important genus of marine bacteria, has significant value in marine antifouling and anticorrosion applications. The utilization of mutator strains has played a crucial role in developing and selecting robust strains with advantageous traits for various biotechnological processes. In this study, by using a combination of whole-genome deep-sequencing and genetic manipulation techniques, we establish a connection between mutator-driven hot mutation loci and their corresponding physiological effects in *Pseudoalteromonas lipolytica *, thereby offering a valuable repository of genetic attributes linked to phenotypic traits in marine bacteria.

## Introduction

The DNA mismatch repair (MMR) system plays a key role in preventing mutation during the DNA replication and recombination process [1]. The major components of the MMR system include MutS, MutL, MutH and UvrD proteins. Among them, MutS acts as a core mismatch recognition protein that recognizes a base–base mismatch or small nucleotide insertion/deletion [2]. Germline mutations of the human MMR gene are the major cause of many cancers, such as Lynch syndrome and cancer of the stomach [3, 4]. In microorganisms, bacteria with a defective MMR system can rapidly evolve by introducing more mutations into their genome, resulting in a mutator phenotype [5]. The occurrence of mutator strains is relatively common in environmental and clinical populations, as well as in experimentally evolving populations of model pathogens, such as *Pseudomonas aeruginosa* and *Vibrio cholerae* [6, 7]. It has been estimated that bacterial species contain mutators within natural populations at varying levels of prevalence. For example, the prevalence of mutators from chronic infections of cystic fibrosis patients is significantly higher at a rate of nearly 90 and 40 % in *Pseudomonas aeruginosa* and *Burkholderia cepacia *, respectively [8, 9]. Among the clinical isolates exhibiting a mutator phenotype, the majority have been attributed to point mutations within the *mutS* gene, indicating that the *mutS* gene plays a crucial evolutionary role in regulating the emergence of the hypermutable phenotype in bacteria.

Since introducing large numbers of mutations can be harmful, bacteria often balance a trade-off between genetic innovation and genetic fidelity to achieve a compromise in bacterial fitness [10]. To improve fitness in specific environmental niches, *mutS*-deficient strains often introduce further mutations in specific targets to change their phenotypic characteristics, such as increasing or decreasing polysaccharide production, as well as resistance to different types of antibiotics [11, 12]. For example, mutations in *mutS* tend to produce a small proportion of mucoid variants of *Pseudomonas aeruginosa* due to mutation of the preferential target *mucA*, which leads to the overproduction of alginate and thus increases virulence [13]. Additionally, inactivation of *mutS* in *Pseudomonas aeruginosa* also significantly increased resistance to ciprofloxacin and rifampicin, mainly by introducing point mutations in *gyrAB*, which encodes DNA gyrase, or *rpoB*, which encodes the β-subunit of RNA polymerase [14, 15]. It has been demonstrated that the genes associated with biofilm formation and flagellar movement are highly susceptible to mutation under specific environmental conditions, resulting in a significant impact on biofilm formation and swimming motility [16, 17]. Indeed, variations in biofilm formation and swimming motility were observed in the Δ*mutS* subpopulation of model pathogens [18]. However, there is still limited knowledge regarding mutator-driven diversification in marine bacteria.

*Pseudoalteromonas* is an important genus of marine bacteria that is widely distributed in various marine environments. It has gained considerable attention due to its ecological significance by producing a large number of active compounds [19, 20]. In a previous study, we demonstrated that the motile marine bacterium *Pseudoalteromonas lipolytica* produced different genetic variants during biofilm formation, such as wrinkled, pigment production and translucent colony morphology variants, which had significant marine antifouling and anticorrosion applications [21–26]. The alteration of colony morphology is often tightly linked to the bacterial mutator phenotype [27]. To investigate mutator-driven diversification in *P. lipolytica *, we first constructed a *mutS*-deficient strain and examined its phenotypic characteristics. Subsequently, we performed whole-genome deep-sequencing on the wild-type *P. lipolytica* and its *mutS* deletion strain to investigate the genetic basis of the mutator-driven diversification. Furthermore, based on analysis of the genome sequencing data, we conducted a genetic manipulation in *P. lipolytica* to experimentally verify the molecular basis of the mutator-driven phenotype. Overall, the results presented in this study established a connection between mutator-driven hot mutation loci and their corresponding physiological effects in *P. lipolytica *.

## Methods

### Strains and growth conditions

The bacterial strains used in this study are listed in Table S1 (available in the online version of this paper). All bacterial strains were stored at −80 °C, and the frozen stocks were streaked onto fresh agar plates prior to experimentation. Assays were conducted using three independent cultures of each strain. *Escherichia coli* WM3064 were grown in LB supplemented with 0.3 mM 2,6-diamino-pimelic acid (DAP) at 37 °C. *P. lipolytica* SCSIO 04301 and its mutant strains were grown in 2216E, SWLB (seawater LB, 1 % tryptone and 0.5 % yeast extract dissolved in seawater) or HSLB (high salt LB, LB medium with 3.4 % salt) at 30 °C. Kanamycin (50 µg ml^−1^) and erythromycin (25 µg ml^−1^) were used to maintain the gene knockout vector pK18mob*sacB*-ery in *E. coli* and *P. lipolytica *, respectively, while chloramphenicol (30 µg ml^−1^) was used to maintain the expression vector pBBR1MCS-cm in both *E. coli* and *P. lipolytica *.

### Construction of gene deletion mutants and expression vectors

Gene deletion mutants of *P. lipolytica* were constructed according to previous methods [28]. The primers used for gene deletion and gene expression are listed in Table S2. Briefly, the upstream and downstream regions of the target gene ORF were PCR-amplified from the wild-type genomic DNA. Two fragments were then ligated into the suicide plasmid pk18mob*sacB*-ery to create the deletion vector in the host *E. coli* WM3064 and then transferred into the chromosome of the wild-type *P. lipolytica* strain by bacterial conjugation. Deletion mutants were generated by screening for erythromycin resistance followed by *sacB*-based counterselection. To construct the expression vectors, the target gene with its native promoter was PCR-amplified and ligated into the plasmid pBBR1MCS-cm in the host *E. coli* WM3064. For *AT00_17115* and *fliP*, the promoters were not directly linked with its ORF. We thus amplified the promoter of *AT00_ 20800*, a constitutive gene encoding glutamine synthetase, and ligated to the intact ORF of AT00_17115 and FliP. The constructed expression vector was then transferred into *P. lipolytica* by bacterial conjugation. The resulting deletion mutants and expression vectors were confirmed by sequencing using the primer sets pK18-f/pK18-r and pBBR1MCS-f/pBBR1MCS-r, respectively (Table S2).

### Attached biofilm

Attached biofilm formation was evaluated by growing the strains in glass tubes with crystal violet staining as reported previously [29]. Briefly, cells were inoculated into 3 ml 2216E medium with an initial turbidity at 600 nm of 0.05 and incubated for 3 days at 25 °C without shaking. The cultures were then removed and the attached biofilm was stained with crystal violet for 20 min. Crystal violet was removed and then rinsed three times using running water. The attached crystal violet was finally dissolved by alcohol for 30 min and the dissolved crystal violet was measured at an absorption of 540 nm. For cell aggregate assays, cells were grown overnight in 2216E medium and then examined under a microscope after crystal violet staining.

### Colony biofilm

Cells were grown overnight in 2216E medium at 25 °C. A 10 µl volume of the cultures was plated on different agar media for different periods of time. Colony biofilms were imaged using a stereoscopic microscope.

### Swimming motility

Cells were grown overnight in 2216E medium at 25 °C. A 1 µl volume of the cultures was inoculated onto 2216E plates containing 0.25 % agar and incubated for 12 h at room temperature. The plates were then imaged and the diameters of the swimming zone were measured.

### Antibiotic resistance assay

Cells of different strains were grown overnight in 2216E medium with or without chloramphenicol at 25 °C. The cultures were then serially diluted 10-fold. A 10 µl volume of the diluted cultures was then plated on SWLB agar that contained different concentrations of antibiotics to determine bacterial viability.

### Quantification of the emergence of translucent variants

Cells from colony biofilm or planktonic cultures were collected and mixed evenly in 1 ml of seawater using 10-fold serial dilutions and subsequently plated on SWLB agar plates for 1 day to obtain about 30–300 colonies per plate. The percentage emergence of the translucent variants was calculated by dividing the total colonies by those with translucent morphology, conducted as previously reported [29]. Three independent experiments were performed, and at least 500 colonies in each independent experiment were examined to calculate the rate of translucent variants.

### Bacterial competition assay

Cells of the wild-type and Δ*mutS* strains were grown overnight in 2216E medium at 25 °C. The two overnight cultures were then diluted into the same fresh 2216E medium with the same initial turbidity of 1.0. The mixed populations were co-cultured with shaking conditions at 25 °C. After a set period of time, the culture was diluted and plated on SWLB agar to obtain about 30–300 colonies per plate, in which at least 500 colonies were examined to calculate the percentage of the Δ*mutS* strains in the co-cultured population based on noticeable differences in colony morphology between the two strains. Assays were performed with two independent cultures of each strain.

### Whole-genome deep-sequencing

Cells of *P. lipolytica* wild-type and Δ*mutS* strains were grown in 2216E medium for 3 days at 25 °C. The cultures were collected and total DNA was extracted using a TIANamp Bacterial DNA Kit (Tiangen). Whole-genome sequencing and mutation analysis were performed by GENEWIZ. The genome was sequenced using BGISEQ-500 sequencing technology and the sequenced reads were assembled using SOAPdenovo v2.04 software. SNPs and indels were detected based on the aligned result of the reference genome of *P. lipolytica* (PRJNA739013).

### Statistical analysis

Statistical analyses were performed using GraphPad Prism v.8.0 software. Data are presented as mean±sd of three independent cultures. Statistical significance was assessed using a two-tailed unpaired Student’s t-test and asterisks are used to represent statistically significant differences [30].

## Results

### Deletion of *mutS* resulted in a mutator phenotype in *P. lipolytica*

**Fig. 1.**
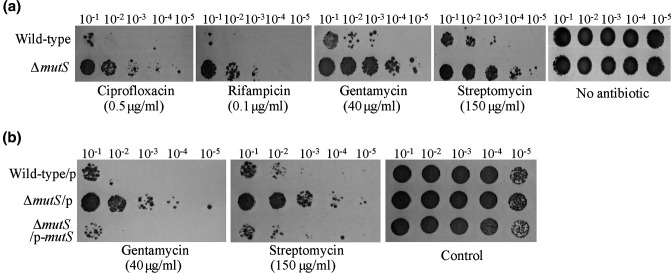
Deletion of *mutS* resulted in a mutator phenotype in *P. lipolytica *. (**a**) The survival of cells was examined after exposure to sublethal concentrations of different antibiotics for the wild-type and Δ*mutS* strains. (**b**) The survival of cells was examined after exposure to sublethal concentrations of gentamicin and streptomycin for the wild-type and Δ*mutS* strains expressing either an empty vector pBBR1MCS (p) or a complementation vector pBBR1MCS-*mutS* (p-*mutS*). Data are from three independent cultures. Images shown in Figs 1–4 are representative images.

To determine the presence of the homologue of MutS protein in *P. lipolytica *, a protein blast search was conducted in the NCBI database using the reference MutS proteins of *E. coli* K-12 and *Pseudomonas aeruginosa* PAO1. As a result, the amino acid sequence of AT00_10435 (or AT00_RS09980) in *P. lipolytica* showed the highest similarity of 66 and 56 % to those of MutS in K-12 and PAO1, respectively. Thus, we named the *AT00_10435* gene *mutS*, and removed it from the genome of *P. lipolytica* using the homologous recombination method (Fig. S1). Mutator strains that exhibit higher mutation rates have often been estimated by determining its resistance to ciprofloxacin or rifampicin antibiotics [31]. To verify the mutator phenotype of the Δ*mutS* strain in *P. lipolytica *, a series of dilution cultures of the wild-type and Δ*mutS* strains were plated on SWLB agar plates containing ciprofloxacin or rifampicin antibiotics at sublethal concentrations of 0.5 and 0.1 µg ml^−1^, respectively. The results showed that the absence of *mutS* increased resistance to both ciprofloxacin and rifampicin by approximately 100-fold compared to the wild-type strain (Fig. 1a). In addition, we found that the absence of *mutS* also led to increased resistance to the aminoglycoside antibiotics (including gentamycin and streptomycin) at a sublethal concentration (Fig. 1a). To further confirm that the increased resistance to antibiotics resulted from the mutation in *mutS*, the complementation plasmid pBBR1MCS-*mutS* was constructed and expressed in Δ*mutS*. As shown in Fig. 1(b), complementation of *mutS* restored the susceptibility to sublethal concentrations of gentamycin and streptomycin in the Δ*mutS* host, whereas expression of the empty plasmid failed to restore the wild-type phenotype (Fig. 1b). Thus, deletion of *mutS* in *P. lipolytica* leads to a mutator phenotype.

### *P. lipolytica* mutator showed changes in biofilm formation and swimming motility

**Fig. 2.**
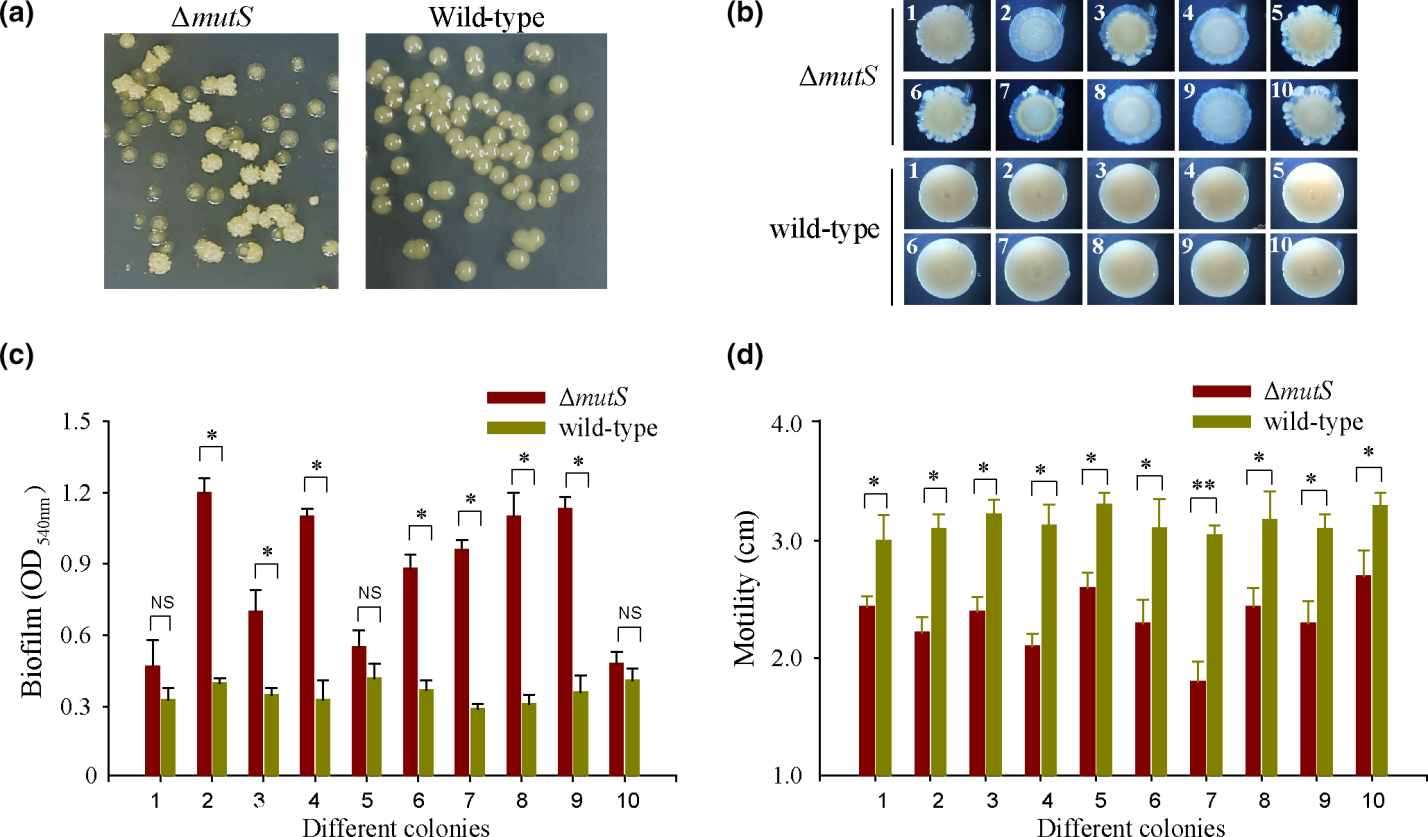
The *P. lipolytica* mutator shows changes in biofilm formation and swimming motility. (**a**) Single colony morphology was examined for the Δ*mutS* and wild-type strains after culture in 2216E medium with shaking for 3 days. (**b**) Colony biofilms were examined for ten randomly picked colonies of the Δ*mutS* and wild-type strains after culture with shaking for 3 days. Attachment of biofilm (**c**) and swimming motility (**d**) was examined for ten randomly picked colonies of the wild-type and Δ*mutS* strains after culture with shaking for 3 days, respectively. Data are shown as mean±sd. Each pair of groups was compared using an unpaired Student’s t-test with GraphPad Prism software, and statistical significance (*P<0.05; **P<0.01; NS, not significant) is indicated.

To explore the phenotypic change in the *P. lipolytica* mutator under passage cultivation, the wild-type and Δ*mutS* strains were cultured in 2216E medium at 30 °C with shaking at 180 r.p.m. for 3 days. The cultures were then serially diluted and plated on agar plates. As shown in Fig. 2(a, b), colonies of Δ*mutS* subcultured cells exhibited a distinctly irregular colony morphology, while all colonies of the wild-type subpopulation strain remained with a smooth and opaque colony morphology (more than 1000 colonies were examined). Ten of the subculture colonies of Δ*mutS* and wild-type strains were then randomly selected for biofilm formation and swimming motility assays. The results showed that all of the wild-type subculture colonies formed a similar attachment biofilm and also showed similar swimming motility on soft agar plates (Fig. 2c. d). However, more than half of the Δ*mutS* subculture colonies showed significantly increased biofilm formation by nearly 2- to 4-fold compared to those of the wild-type strains. (Fig. 2c). In contrast, all of the Δ*mutS* subculture colonies showed reduced swimming motility to various degrees (Fig. 2d). These results indicated that the Δ*mutS* subpopulation has high phenotypic diversity.

### *P. lipolytica* mutator produces a high proportion of translucent colony morphology variants

**Fig. 3.**
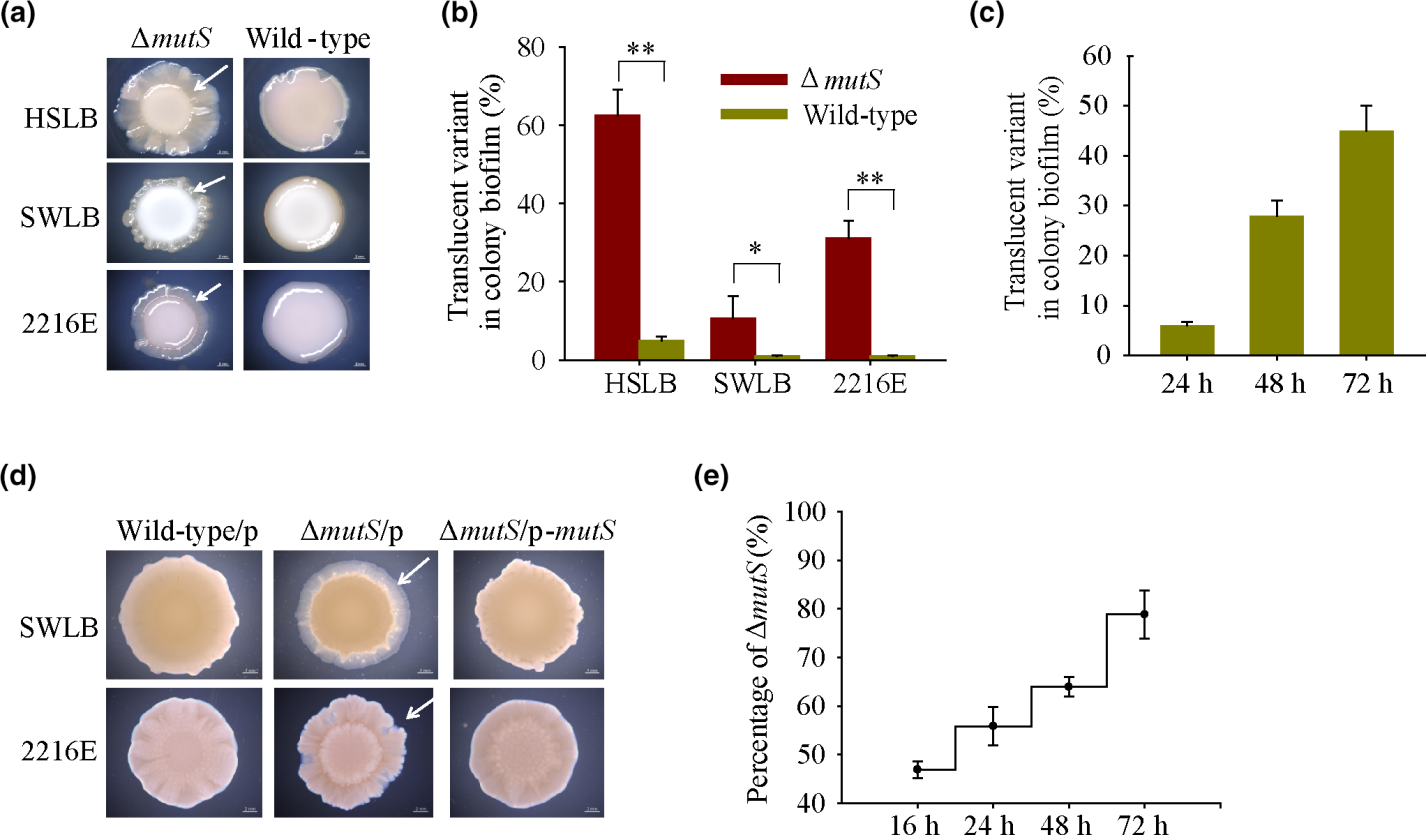
*
P. lipolytica* mutator produces a high proportion of translucent colony morphology variants. (**a**) Generation of the translucent variants was examined for the wild-type and Δ*mutS* strains incubated on different media agar plates for 3 days. (**b**) Production rates of the translucent variants were measured for the wild-type and Δ*mutS* strains incubated on different media agar plates for 3 days. (**c**) Production rates of the translucent variants were measured by culturing the Δ*mutS* strain under shaking conditions at 30 °C for different periods of time. (**d**) Colony biofilms were examined for the wild-type and Δ*mutS* strains expressing either the empty vector pBBR1MCS (p) or the complementation vector pBBR1MCS-*mutS* (p-*mutS*). Arrows indicate the production of translucent variants. (**e**) Competitive fitness of Δ*mutS* against the wild-type strain was examined for different periods of time. Data are shown as mean±sd. Each pair of groups was compared using an unpaired Student’s t-test with GraphPad Prism software, and statistical significance (*P<0.05; **P<0.01) is indicated.

We have demonstrated previously that translucent variants isolated from wild-type *P. lipolytica* grown on HSLB agar plates showed enhanced biofilm formation [29]. To examine the colony morphologies of the *P. lipolytica* mutator strain, we plated 10 µl of the overnight cultures of the Δ*mutS* strain on HSLB, SWLB and 2216E agar plates. After incubation at 25 °C for 3 days, variants with a translucent morphology began to emerge in a large proportion at the edge of the colonies (Fig. 3a). In contrast, no translucent morphology variants were observed for the wild-type strain on either SWLB or 2216E agar plates (Fig. 3a), while a small proportion of translucent variants were observed on HSLB agar plates, as we reported previously [29]. We next calculated the percentages of the translucent variants that evolved from the ancestor of the Δ*mutS* strain. As shown in Fig. 3(b), the proprotions were 62±7, 31±5 and 11±6 % on HSLB, 2216E and SW-LB agar plates, respectively. In addition, when the Δ*mutS* strain was cultured on 2216E under shaking conditions, the production rate of the translucent variants increased from 6±2 % at day 1 to 45±7 % at day 3 (Fig. 3c). To further verify that the emergence of translucent variants was directly linked to the deletion of *mutS*, we complemented *mutS* in the Δ*mutS* strain via plasmid pBBR1MCS-*mutS* under the control of its own promoter. As expected, ectopic expression of MutS in the Δ*mutS* strain produced very few translucent variants in the ageing colony. In contrast, expression of the empty plasmid pBBR1MCS in the Δ*mutS* strain still produced translucent morphology variants on SWLB or 2216E agar medium (Fig. 3d). A previous study showed that alterations in colony morphology for an evolving population are often associated with adaptation fitness [32]. Thus, we examined the competitive fitness of Δ*mutS* against the wild-type strain by growing co-cultures of the two strains with the same initial inoculum for different periods of time in 2216E medium. As shown in Fig. 3(e), Δ*mutS* cells accounted for 80±5 % after 72 h of incubation, suggesting that Δ*mutS* had a fitness advantage compared to the wild-type strain. Together, these results demonstrated that the *P. lipolytica* mutator strain increased cell fitness and produced a high proportion of translucent colony morphology variants during the growth period.

### Whole-genome re-sequencing of the Δ*mutS* whole population

**Fig. 4.**
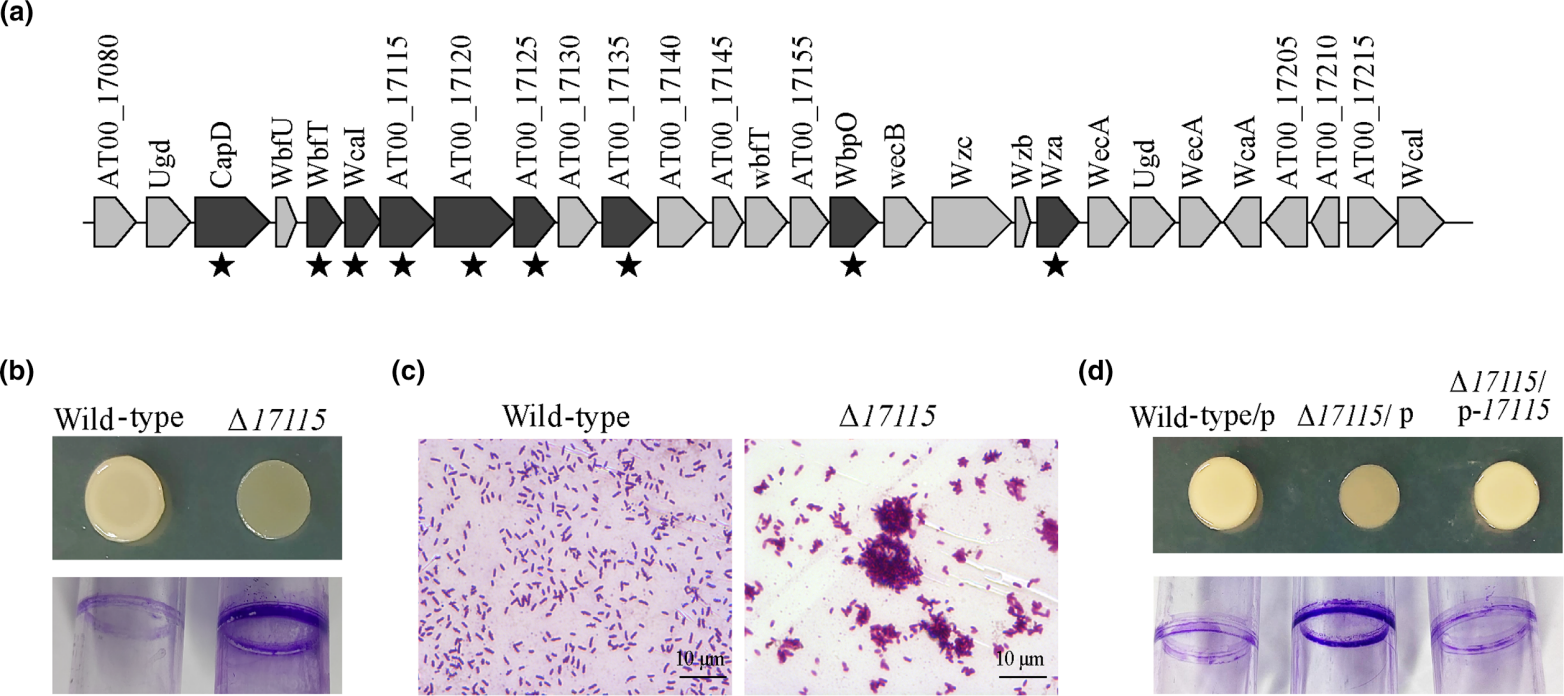
Deletion of the *AT00_17115* gene resulted in a translucent colony morphology. (**a**) The capsular polysaccharide biosynthesis cluster of *P. lipolytica* ranges from *AT00_17080* to *AT00_17220*. Asterisks indicate the hot insertion sites within the *cps* operon during colony biofilm formation. (**b**) Colony biofilms and attachment biofilms were examined for the wild-type and Δ*AT00_17115* strains. (**c**) Cell aggregates were examined for the wild-type and Δ*AT00_17115* strains under a microscope. (**d**) Colony biofilms and attachment biofilms were examined for the wild-type and Δ*AT00_17115* strains expressing either the empty vector pBBR1MCS (p) or the complementation vector pBBR1MCS-*17115S* (p-*17115*).

To explore the mutation genes that are responsible for the observed phenotypes in the Δ*mutS* strain, the wild-type and Δ*mutS* strains were cultured in 2216E medium with shaking for 3 days, under which translucent variants were distinctly produced in the Δ*mutS* strain (Fig. 3c). Bacterial populations of the two strains were collected, and the genomic DNAs were extracted to perform whole-genome re-sequencing. Statistical analyses of the whole-genome sequencing data of the two strains are presented in Table S3. Sequencing coverage of the two strains reached 99.8 % and the sequencing depths were 468× and 609× for the wild-type and Δ*mutS* strains, respectively. By aligning the sequences to the wild-type genome, 37 sites of mutation (designated M1–M37) were identified only in the genome of the Δ*mutS* population (Table 1). Given that the Δ*mutS* strain only produced a certain proportion of the translucent variants during the growth period, we hypothesized that the mutation target associated with the emergence of phenotypic variants must exhibit a specific mutation rate in a particular gene. Of the 37 mutations observed, five mutations located at the *AT00_02985*, *AT00_08855*, *AT00_16175*, *AT00_17115* and *AT00_*18850-*AT00_*18860 intergenic regions showed a specific mutation rate, indicating these mutations occurred only in a fraction of cells within the Δ*mutS* population. Notably, one of these mutations, a G duplication (153dupG), was identified in the coding region of the *AT00_17115* gene, with a mutation rate of 31 %. The *AT00_17115* gene is predicted to encode a heparinase within the *cps* operon (Fig. 4a, from *AT00_17080* to *AT00_17220*), which is responsible for the synthesis of the capsular polysaccharide (CPS) to maintain an opaque colony morphology [29]. Hence, the identified mutation in the *AT00_17115* gene may be accountable for the emergence of translucent variants in the Δ*mutS* population during the growth period. Furthermore, as the Δ*mutS* subculture colonies exhibited reduced swimming motility, we hypothesized that the mutator strain may introduce mutations in specific genes located within the flagellar biosynthesis cluster. As expected, we found that two genes, *AT00_08855* and *AT00_09085*, that are involved in flagellar biosynthesis harbour non-synonymous point mutations in their coding region, which may be associated with the alteration of swimming motility in the Δ*mutS* subpopulation.

**Table 1.** Mutations in the Δ*mutS* population identified by whole-genome re-sequencing

No.	Gene	Product	Genome position	Change in DNA	Change in protein	Rate
M1	AT00_00860	Serine hydroxymethyltransferase	189 297	A341G	D114G	1
M2	AT00_01155	Chloride channel protein	249 164	T1424C	I475T	1
M3	AT00_01285	Ubiquinol oxidase subunit II	276 866	622delG	Frameshift	1
M4	AT00_01765	Alkylhydroperoxidase	378 285	G263A	R88H	1
M5	AT00_01975	Fucose permease	429 748	T947C	M316T	1
M6	AT00_02205	Mechanosensitive ion channel	485 155	A523G	T175A	1
M7	AT00_02365	Capsule biosynthesis protein CapB	529 306	T497C	L166S	1
M8	AT00_02985	Hypothetical protein	660 360	G280A	E94K	0.46
M9	AT00_03160	Apolipoprotein *N*-acyltransferase	699 038	T605C	V202A	1
M10	AT00_03380	Hydroxyacylglutathione hydrolase	734 550	T526C	F176L	1
M11	AT00_03505	Diguanylate cyclase	754 336	A2171G	N724S	1
M12	Intergenic		964 383			1
M13	AT00_05405	ATP-binding protein	1 165 812	C598T	R200C	1
M14	AT00_06005	Cell division protein ZapC	1 301 001	A359G	E120G	1
M15	AT00_06730	YciI family protein	1 466 917	A103G	N35D	1
M16	AT00_07185	Hypothetical protein	57 815	G103A	D35N	1
M17	AT00_07540	Hypothetical protein	143 918	A284G	Q95R	1
M18	AT00_08855	Flagellar ATPase synthesis	417 727	C52G	H18D	0.52
M19	AT00_09085	Flagellar protein FlgP	484 848	A455G	Y152C	1
M20	AT00_10020	DEAD/DEAH box helicase	684 923	G919A	G307S	1
M21	AT00_10245	Pantoate-beta-alanine ligase	736 997	T574C	Y192H	1
M22	Intergenic		744 855			1
M23	AT00_10695	Acetylglutamate kinase	845 179	C659T	A220V	1
M24	AT00_10890	Purine metabolism	882 529	A3203G	D1068G	1
M25	AT00_12615	Alpha/beta hydrolase	86 527	A101G	N34S	1
M26	AT00_13635	Chemotaxis protein	311 678	A176G	D59G	1
M27	AT00_13740	TonB-dependent receptor	333 260	T1933C	F645L	1
M28	Intergenic	Hypothetical protein	862 871			1
M29	AT00_16175	FHA domain-containing protein	10 952	C37T	P13S	0.49
M30	AT00_17115	Heparinase family protein	224 049	153dupG	Frameshift	0.31
M31	AT00_17785	Metallopeptidase M24 family	372 898	T277C	F93L	1
M32	AT00_18255	Metallopeptidase M1 family	461 420	G47A	C16Y	1
M33	AT00_18800	Glutamine amidotransferase	598 608	C467T	A156V	1
M34	Intergenic		609 338			0.22
M35	AT00_19130	F0F1 ATP synthase subunit	663 155	G763T	D255Y	1
M36	AT00_19270	Hypothetical protein	23 202	C589T	Q197X	1
M37	AT00_19445	Hypothetical protein	59 174	A31G	T11A	1

### Deletion of the *AT00_17115* gene resulted in a translucent colony morphology

We previously reported that an insertion sequence (IS) element inserted into the *cps* operon at multiple sites during biofilm formation in *P. lipolytica* (asterisks in Fig. 4a), including the *AT00_17115* gene, can lead to the production of translucent variants due to a reduction in CPS production [29]. To further verify that disruption of the *AT00_17115* gene gave rise to the translucent colony morphology variants in the Δ*mutS* population, we deleted the *AT00_17115* gene and investigated the physiological functions of the corresponding mutant strain (Fig. S2). As expected, the Δ*AT00_17115* strain also showed translucent colony morphology (Fig. 4b). Moreover, deletion of the *AT00_17115* gene significantly increased the formation of attachment biofilm (Fig. 4b) and cell aggregates (Fig. 4c). To further verify the physiological function of *AT00_17115*, the complementation plasmid pBBR1MCS-*17115* was constructed and expressed in Δ*17115*. As shown in Fig. 4(d), complementation of *AT00_17115* restored the opaque colony morphology and also formed thin attachment biofilm, whereas expression of the empty plasmid failed to restore the wild-type phenotype (Fig. 4d). Together, this demonstrates that the spontaneous mutation in the *AT00_17115* gene within the *cps* operon resulted in the production of translucent variants in the Δ*mutS* population.

### Deletion of the *AT00_08855* and *AT00_09085* genes caused a reduction in swimming motility

**Fig. 5.**
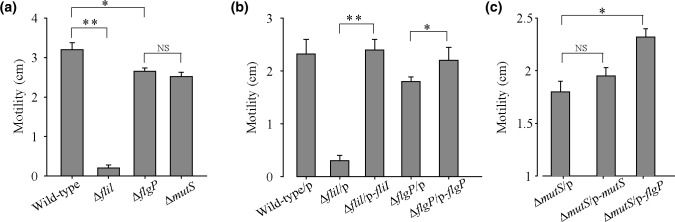
Deletion of the *AT00_08855* and *AT00_09085* genes caused a reduction in swimming motility. (**a**) Swimming motility was examined for the wild-type, Δ*fliI*, Δ*flgP* and Δ*mutS* strains. (**b**) Swimming motility was examined for the wild-type, Δ*fliI* and Δ*flgP* strains expressing either an empty vector or a complementation vector. (**c**) Swimming motility was examined for the Δ*mutS* strains expressing either an empty vector or a complementation vector p-*mutS* and p-*flgP*. Data are shown as mean±sd. Each pair of groups was compared using an unpaired Student’s t-test with GraphPad Prism software, and statistical significance (*P<0.05; *P<0.01; NS, not significant) is indicated.

Based on whole-genome re-sequencing, we found that point mutations in *AT00_08855* and *AT00_09085* located within the flagellar biosynthesis cluster may be associated with altered swimming motility in the Δ*mutS* subpopulation. AT00_08855 in *P. lipolytica* shares 65 % similarity (98 % coverage) with the FliI protein of *V. cholera*, which is a flagellum-specific ATPase, while *AT00_09085* shares 42 % similarity (86 % coverage) with the FlgP protein of *V. cholera*, which is required for the assembly of a stable flagellum. The *fliI* gene was identified with a non-synonymous point mutation (C52G) in the coding region at a frequency of 52 % in the Δ*mutS* subpopulation, suggesting *fliI* is a hot mutation target during the growth period in the mutator strain. To confirm the physiological function of the *fliI* gene, an in-frame deletion of the *fliI* gene was constructed in wild-type *P. lipolytica* (Fig. S3). As shown in Fig. 5(a), deletion of *fliI* greatly inhibited swimming motility (Fig. 5a). We further expressed FliI via the pBBR1MCS-*fliI* plasmid in the Δ*fliI* strain. The results showed that induction of FliI fully restored swimming motility in the Δ*fliI* strain, whereas the empty plasmid pBBR1MCS failed to do so, suggesting the *fliI* gene plays an important role in swimming motility (Fig. 5b). Regarding *flgP*, a non-synonymous point mutation (A455G) was identified in the coding region at a frequency of 100 % in the Δ*mutS* subpopulation, suggesting that the original Δ*mutS* strain used in this study may have already carried a mutation in the *flpP* gene. To verify this, we first deleted the *flpP* gene in the wild-type *P. lipolytica* and subsequently assessed its swimming motility (Fig. S4). As expected, both the Δ*flgP* and Δ*mutS* strains showed similar motility activity, which was weaker than that of the wild-type strain (Fig. 5a). We then expressed FlpP via the pBBR1MCS-*flgP* plasmid in the Δ*flgP* and Δ*mutS* strains. The results showed that expression of FlgP restored swimming motility in the Δ*flgP* strain, while expression of the empty plasmid pBBR1MCS failed to function (Fig. 5b). In addition, ectopic expression of FlgP in the Δ*mutS* strain also restored swimming motility, whereas expression of the empty plasmid pBBR1MCS or pBBR1MCS-*mutS* was ineffective (Fig. 5c). Together, these findings demonstrated that the reduced swimming motility in the Δ*mutS* subpopulation was caused by the introduction of spontaneous mutations in the *fliI* and *flgP* genes in *P. lipolytica *.

## Discussion

**Fig. 6.**
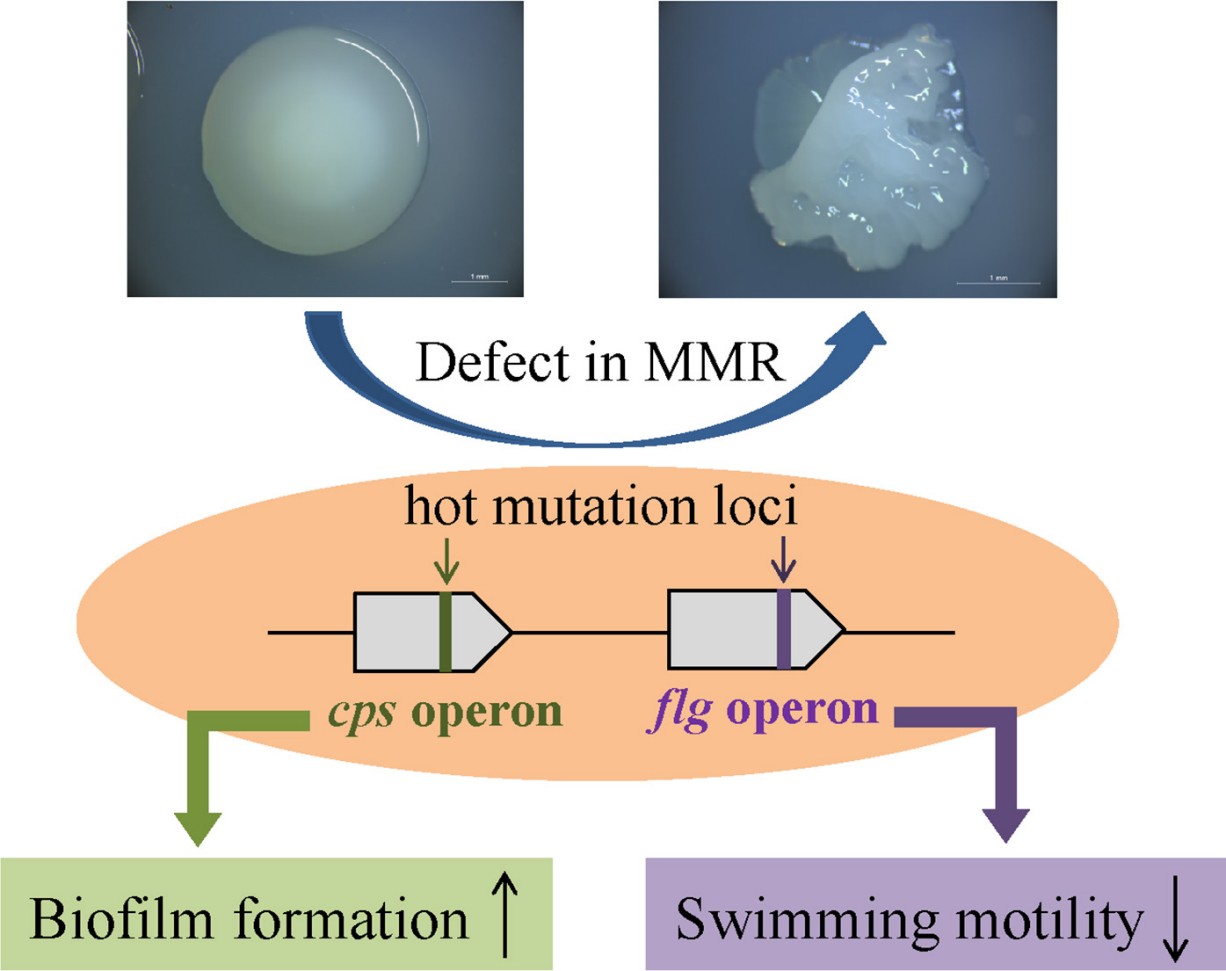
Summary of the genetic and phenotypic variations in the *P. lipolytica* mutator strain. *P. lipolytica* exhibited irregular colony morphology due to defects in the mismatch repair (MMR) system. The mutator strain preferentially introduces mutations located at the *cps* operon and flagellar operon, resulting in increased biofilm formation but inhibited swimming motility.

To gain better insight into the evolution of genetic variants in the *P. lipolytica* mutator strain, we summarized the mutator-driven hot mutation loci and their corresponding physiological effects in *P. lipolytica* (Fig. 6). *P. lipolytica* defects in the MMR system showed a mutator phenotype and exhibited high phenotypic diversity in terms of colony morphology, biofilm formation and swimming motility. Importantly, we found that the mutator strain preferentially introduces the mutations located at the *cps* operon and flagellar operon, which leads to an increase in biofilm formation but an inhibition of swimming motility. Although the mutations result in decreased CPS biosynthesis and motility activity, bacteria can effectively reduce energy consumption and also enhance biofilm formation to obtain better adaptation fitness in specific environmental niches. Thus, study of the mutator of *P. lipolytica* provides insights into genetic evolution linked to microbial fitness.

Deficiencies in the MMR system allow bacteria to rapidly evolve and improve fitness under different environmental conditions [33]. However, there is still limited knowledge regarding mutator-driven evolution in marine bacteria. Here, we investigated mutator-driven diversification and its molecular basis in the marine *P. lipolytica *. First, deletion of *mutS* in *P. lipolytica* significantly increased the mutation rates by approximately 100-fold. Furthermore, variants within the Δ*mutS* subpopulation exhibited increased biofilm formation but reduced swimming motility, suggesting that genetic changes occurred during the growth period. Whole-genome re-sequencing in the Δ*mutS* population, combined with genetic manipulation, revealed that spontaneous mutation in the *AT00_17115* gene within the *cps* operon resulted in the emergence of translucent variants, thereby promoting enhanced biofilm formation. Additionally, point mutations in the *fliI* and *flgP* genes within the flagellar cluster were also identified in the Δ*mutS* subpopulation, which caused a reduction in swimming motility. Together, this study provides a molecular basis for the diversification driven by the *P. lipolytica* mutator strain, specifically in relation to biofilm formation and swimming motility in marine *P. lipolytica *.

Pathogenic bacteria equipped with an intact CPS showed significantly increased resistance to host antimicrobials, hospital disinfectants and desiccation [34]. In marine bacteria, an alteration in CPS production is often associated with changes in colony morphology, where opaque colonies can spontaneously become translucent due to reduced CPS production, such as in *Vibrio parahaemolyticus* and *Vibrio vulnificus* [35–37]. It has been reported that inactivation of *mutS* in *V. parahaemolyticus* also produces a number of translucent variants that evolved from the wild-type strain with opaque colony morphology, ranging from 28 to 100 % after overnight culturing, but the molecular basis of the specific mutation target remains unknown [38]. In the present study, the *P. lipolytica mutS* mutant strain evolved translucent variants at a rate exceeding 60 % under laboratory culture conditions, suggesting that CPS expression is a hot regulatory target when the DNA MMR system is inactivated. In a previous study, we experimentally demonstrated that translucent variants of *P. lipolytica* found in biofilm are a direct consequence of a spontaneous point mutation or IS*5* insertion in any one of the nine genes within the *cps* operon (*AT00_17080* to *AT00_17220*) [21, 29]. Here, through whole-genome deep-sequencing we identified a Δ*mutS* population harbouring a mutation in the *AT00_17115* gene within the *cps* operon at a frequency of 31 %. However, no mutations were detected in the other genes within the *cps* operon. This could potentially be attributed to the limitation of the culture conditions, which favour the enrichment of a specific translucent variant with one particular mutation in the *cps* operon.

Flagellar motility is a crucial factor for bacterial survival in fluctuating environments. However, genes involved in flagellar biosynthesis are often hot mutation targets under specific environmental conditions, which allows bacteria to inhibit motility and reduce the consumption of energy [39]. For example, during long-term colonization in adult mice, spontaneous non-motile variants of *V. cholerae* were highly produced at a rate of approximately 10 %, in which the mutations occurred primarily in conserved regions of the flagellar regulatory genes *flrA* and *flrC* [40]. We previously also found that wrinkled variants isolated from *P. lipolytica* biofilms harbour spontaneous mutations in the flagellar regulatory gene *wspF* or flagellar biosynthesis gene *flhA*, leading to a reduction in swimming activity [21, 25]. However, there is still limited knowledge linking reduced swimming motility to the specific hot mutation targets introduced by the *mutS* deletion. In this study, we found that the Δ*mutS* population harbours a non-synonymous mutation in the *fliI* and *flpP* genes. FliI is a flagellum-specific ATPase that interacts with FliH and FliJ to form the transmembrane flagellar export complex in the flagellar type III secretion system (fT3SS). Deletion of *fliI* significantly reduces the protein transport activity of the fT3SS, resulting in an approximately twofold decrease in flagellar filament production and a very weak motile phenotype [41–45]. The gene *flgP* encodes an outer membrane lipoprotein required for the assembly of a stable flagellum [46]. In *V. cholera*, deletion of *flgP* produced fragile and defective flagella, which also resulted in a slightly motile phenotype [47]. Although mutations in *flgP* and *fliI* were identified in the *P. lipolytica* Δ*mutS* population, it remains to be determined whether such mutations occur naturally or under stressful environmental conditions.

The emergence of phenotypic variants due to deficiencies in the MMR system has been shown in many bacterial species. However, specific mutation targets triggered by the MMR that are linked to the occurrence of a mutator-driven phenotype have rarely been identified in marine bacteria. This study provides direct evidence linking mutation of the *AT00_17115* gene with an increase in biofilm formation, and mutations of the *fliI* and *flgP* genes with a reduction in swimming motility in the mutator strain of *P. lipolytica *. The use of mutator strains has been instrumental in the development and selection of robust strains with advantageous traits for various biotechnological processes [48, 49]. Given the significant impact of *P. lipolytica* on marine antifouling and anticorrosion, further endeavours should be undertaken to expand the selection and identification of mutator-derived variants exhibiting potent antifouling and anticorrosion activities.

## Supplementary Data

Supplementary material 1
