# Supplementary material for: Molecular basis of the phenotypic variants arising from a Pseudoalteromonas lipolytica mutator
Source: Microb Genom. 2023 Oct 18;9(10):001118. doi: 10.1099/mgen.0.001118 (PMC10634453; doi:10.1099/mgen.0.001118)
Supplement: Supplementary material 1 [file mgen-9-1118-s001.pdf]

SUPPLEMENTARY FILE

Molecular basis of the phenotypic variants arose from *Pseudoalteromonas lipolytica* mutator

Zhenshun Zeng[1], Jiayu Gu[2,3], Shituan Lin[2,3], Qian Li[1], Weiquan Wang[2,3] and Yuexue Guo[2,3*]

[1]Key Laboratory for Water Quality and Conservation of the Pearl River Delta, Ministry of Education, School of Environmental Science and Engineering, Guangzhou University, Guangzhou, China

[2]Key Laboratory of Tropical Marine Bio-Resources and Ecology, Guangdong Key Laboratory of Marine Materia Medica, RNAM Center for Marine Microbiology, South China Sea Institute of Oceanology, Chinese Academy of Sciences, Guangzhou, China

[3]University of Chinese Academy of Sciences, Beijing, China

Table S1. Bacterial strains and plasmids used in this study.

Strains and plasmids	Relevant characteristics	Source
Strains		
E.coli WM3064	RP4(tra) in chromosome, DAP-	(Dehio and Meyer 1997)
P. lipolytica	Wild-type strain	(Zeng, et al 2014)
P. lipolytica Δ*mutS*	*mutS* gene deletion mutant	This study
P. lipolytica Δ*17115*	*17115*gene deletion mutant	This study
P. lipolytica Δ*fliI*	*fliI* gene deletion mutant	This study
P. lipolytica Δ*flgP*	*flgP* gene deletion mutant	This study
Plasmids		
pK18mob*sacB*-ery	Gene knockout vector, Kan^r, Ery^r	(Wang et al. 2015)
pK18mob*sacB*-ery-*mut*S	Vector for deleting *mutS*	This study
pK18mob*sacB*-ery-*17115*	Vector for deleting *17115*	This study
pK18mob*sacB*-ery-*fliI*	Vector for deleting *fliI*	This study
pK18mob*sacB*-ery-*flgP*	Vector for deleting *flgP*	This study
pBBR1MCS-cm	Expression vector, Cm^r	(Wang et al. 2015)
pBBR1MCS-*mutS*	Vector for expression *mutS*	This study
pBBR1MCS-*17115*	Vector for expression *17115*	This study
pBBR1MCS-*flpP*	Vector for expression *flpP*	This study
pBBR1MCS-*fliI*	Vector for expression *fliI*	This study

Table S2. Sequences of primers used in this study.

Primer name	Sequence
**In-frame deletions**	
mutS-up-S	GCTCTAGATCACCCATAAAAAAACCGCT
mutS-up-A	AACTGCAGGTATTTACCCAAAGGCTGAA
mutS-down-S	AACTGCAGTTTGACAATGAACCCTCTGA
mutS-down-A	CCCAAGCTTGCCAAAACAACGAGAGTATG
mutS-SF	TTTGCTTGGTTGCTGTATG
mutS-SR	TTGAAGATGTCGGTCGTGA
mutS-LF	CGGTTCAGTGCGACTATTAT
mutS-LR	GCTCACAAAATCGGCACAAA
17115-up-S	GCCAGTGCCAAGCTTGCATGCGCGAAGCAACCACTGATACCC
17115-up-A	CCCAGACCAAACATGAGAGATAGCCCCCCCC
17115-down-S	TCTCTCATGTTTGGTCTGGGTTTCGTGTAGC
17115-down-A	AAGATTAGTCACTGGGGATCCATTTAGGGGTTTTGTAGCGGC
17115-SF	AAGGCATTTGGTTGGAAAG
17115-SR	ATAGGTCTGGGGCATAGTA
17115-LF	GCCCGTAGTTGGTTTTGTTG
17115-LR	TTAAACCTTGCCCTTCGCCT
fliI-up-S	gccagtgccaagcttgcatgcAAGAACCTGAATTTCCACCTTTGA
fliI-up-A	gctgtgccatCCTAATAAGCCCATCCCTACAGG
fliI-down-S	gcttattaggATGGCACAGCGTGAAATAGCG
fliI-down-A	aagattagtcactggggatccAGGCAAACTCGTCCAACATTTT
fliIP-SF	AAGAAGGTGTTGAGCTGGGTA
fliI-SR	AGCCGAGTCAGCAATAGGG
fliI-LF	ATGAACTACCTTGATACCAATATTGAAGG
fliI-LR	GCCTCTGATTCTTTGCTTAAAAAAC
flgP-up-S	acgacggccagtgccaagcttCAGTTTTTCATAGGTTGTGCCATC
flgP-up-A	ATTGGGTAGTCATCTGGTTCAATG
flgP-down-S	gaaccagatgactacccaatTATTGGTGAAATTAAGCCGCG
flgP-down-A	agtcactggggatcctctagaACACTTTAAATACGCTCACCATTTAAC
flgP-SF	TGTGTCAAAACCCCGTCAACA
flgP-SR	GCCATGCCAAAGAACAGAACA
flgP-LF	ATGTAAGATCTTGCCTTGAGTG
flgP-LR	TGCGTTTTATTATGGTGTCG
**Complementation**	
mutS-pBBR-F	ACGCGTCGACGCTCTAACACTGGCTCTATC
mutS-pBBR-R	CCGCTCGAGCAGCTTTTTTTATGCACGCA
17115-pBBR-F1	gtgaccgtgtgcttcgaattcCGATGCTCATATTTTTTCTCAGAGA
17115-pBBR-R1	tGTGTCCTCCAATTGTTGGGTTT
17115-pBBR-F2	cccaacaattggaggacacATGAAACTAACGTTAATCAAGTTATCTAAGA
17115-pBBR-R2	gtcgacggtatcgataagcttTCAATGTATTTCCTTTCTAAACTCTATTTTT
fliIP-pBBR-F1	gtgaccgtgtgcttcgaattcCGATGCTCATATTTTTTCTCAGAGA
fliIP-pBBR-R1	gactactcatGTGTCCTCCAATTGTTGGGTTT

fliIP-pBBR-F2	tggaggacacATGAGTAGTCAATCAATGACCCTTAGC
fliIP-pBBR-R2	gtcgacggtatcgataagcttTTATGCTTGTCCTAATAACTGCGC
flpP-pBBR-F	gtgaccgtgtgcttcgaattcTGTGTCAAAACCCCGTCAACA
flpP-pBBR-R	gtcgacggtatcgataagcttGCCATGCCAAAGAACAGAACA
Sequencing primers	
pK18-f	ATTCCGCTGGCAGCTTAAG
pK18-r	GGTAACGCCAGGGTTTTCC
pBBR1MCS-f	TCGTTAAATAGCCGCTTATG
pBBR1MCS-f	AATTTCACACAGGAAACAGC

Table S3. Statistical analysis of the whole-genome sequencing results using the genome of *Pseudoalteromonas lipolytica* SCISO _04301 (GenBank assembly accession: GCA_000576675.1) as reference.

Samples	Reads length (BP)	Raw data (Mb)	Total reads (bp)	Covered length (bp)	Clean data (Mb)	Coverage (%)	Depth (X)
Wild-type	150	2,366	15,775,578	4,725,785	2, 362	99.83	468
Δ*mutS*	150	4,732	31,698,060	30,271,454	2, 362	99.78	609

Figure S1. In-frame deletion of *mutS* in *P. lipolytica* was confirmed by PCR using four primer sets. M indicates marker. Lanes 1, 3, 5 and 7 used DNA from the wild-type strain, and lanes 2, 4, 6 and 8 used DNA from the *mutS* mutant strain. The expected product sizes were 3009 bp, 4466 bp, 4536 bp and 5993 bp for the wild-type and 549 bp, 2006 bp, 2076 bp and 3533 bp for the Δ*mutS*.

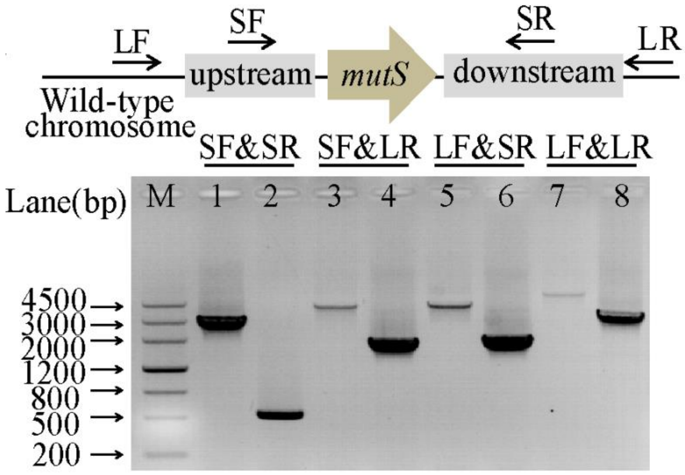

Figure S2. In-frame deletion of *AT00_17115* in *P. lipolytica* was confirmed by PCR using four primer sets. M indicates marker. Lanes 1, 3, 5 and 7 used DNA from the wild-type strain, and lanes 2, 4, 6 and 8 used DNA from the *AT00_17115* mutant strain. The expected product sizes were 1865 bp, 2513 bp, 2646 bp and 3294 bp for the wild-type and 820 bp, 1468 bp, 1601 bp and 2249 bp for the Δ*AT00_17115*.

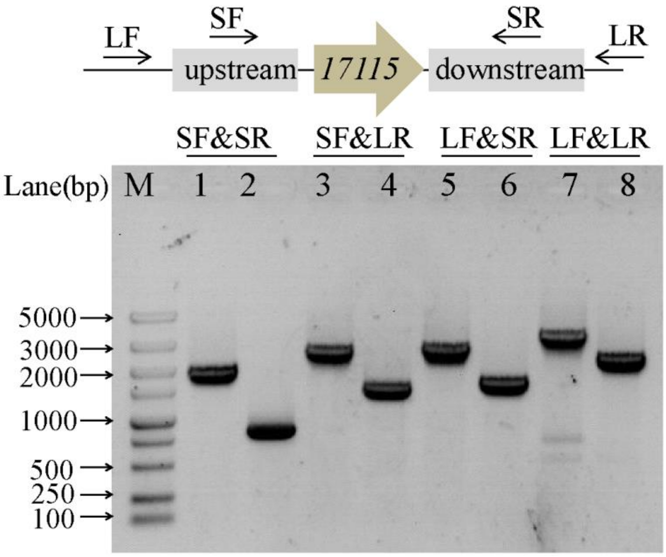

Figure S3. In-frame deletion of *fliI* in *P. lipolytica* was confirmed by PCR using four primer sets. M indicates marker. Lanes 1, 3, 5 and 7 used DNA from the wild-type strain, and lanes 2, 4, 6 and 8 used DNA from the *fliI* mutant strain. The expected product sizes were 1486 bp, 2453 bp, 2225 bp and 3192 bp for the wild-type and 1005 bp, 1972 bp, 1744 bp and 2711 bp for the Δ*fliI*.

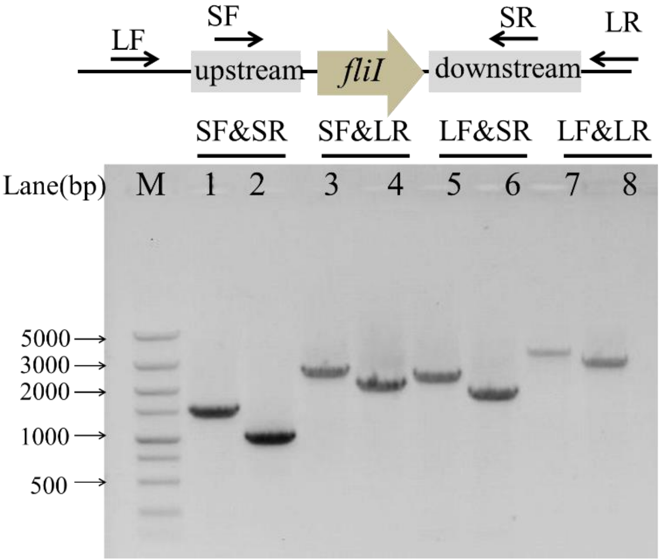

Figure S4. In-frame deletion of *flgP* in *P. lipolytica* was confirmed by PCR using four primer sets. M indicates marker. Lanes 1, 3, 5 and 7 used DNA from the wild-type strain, and lanes 2, 4, 6 and 8 used DNA from the *flgP* mutant strain. The expected product sizes were 922 bp, 1947 bp, 1628 bp and 2653 bp for the wild-type and 625 bp, 1650 bp, 1331 bp and 2356 bp for the Δ*flgP*.

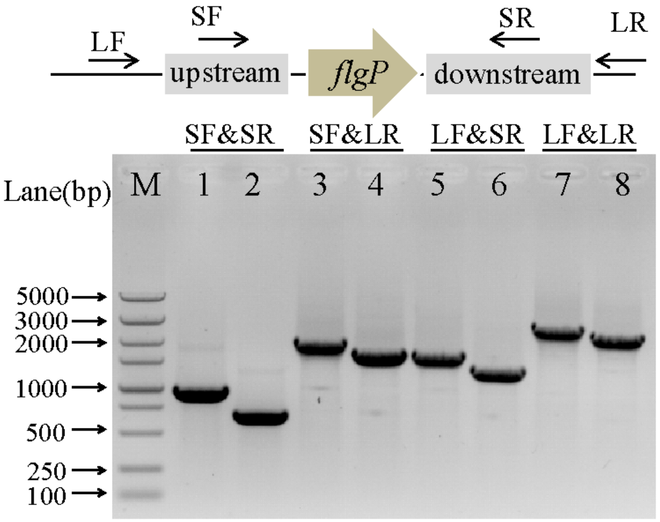

Reference

Dehio C, Meyer M (1997) Maintenance of broad-host-range incompatibility group P and group Q plasmids and transposition of Tn5 in Bartonella henselae following conjugal plasmid transfer from *Escherichia coli*. J Bacteriol 179: 538–540.

Zeng, Z.S., Dai S.K., Xie Y.C., Tian X.P., Li J, and Wang X.X. (2014). Genome sequences of two *Pseudoalteromonas* strains isolated from the South china sea. *Genome Announc* 2: e00305–14.

Wang, P.X., Yu, Z.C., Li, B.Y., Cai, X.S., Zeng, Z.S., Chen, X.L., and Wang, X.X. (2015). Development of an efficient conjugation-based genetic manipulation system for *Pseudoalteromonas*. *Microb Cell Fact* 14: 11.
